# Binge-like sucrose consumption reduces the dendritic length and complexity of principal neurons in the adolescent rat basolateral amygdala

**DOI:** 10.1371/journal.pone.0183063

**Published:** 2017-08-16

**Authors:** Masroor Shariff, Paul Klenowski, Michael Morgan, Omkar Patkar, Erica Mu, Mark Bellingham, Arnauld Belmer, Selena E. Bartlett

**Affiliations:** 1 Institute of Health and Biomedical Innovation at Translational Research Institute, Queensland University of Technology, Brisbane, Australia; 2 School of Biomedical Sciences, The University of Queensland, Brisbane, Queensland, Australia; Radboud University Medical Centre, NETHERLANDS

## Abstract

A compelling body of evidence suggests that the worldwide obesity epidemic is underpinned by excessive sugar consumption, typified by the modern western diet. Furthermore, evidence is beginning to emerge of maladaptive changes in the mesolimbic reward pathway of the brain in relation to excess sugar consumption that highlights the importance of examining this neural circuitry in an attempt to understand and subsequently mitigate the associated morbidities with obesity. While the basolateral amygdala (BLA) has been shown to mediate the reinforcing properties of drugs of abuse, it has also been shown to play an important role in affective and motivated behaviours and has been shown to undergo maladaptive changes in response to drugs of abuse and stress. Given the overlap in neural circuitry affected by drugs of abuse and sucrose, we sought to examine the effect of short- and long-term binge-like sucrose consumption on the morphology of the BLA principal neurons using an intermittent-access two-bottle choice paradigm. We used Golgi-Cox staining to impregnate principal neurons from the BLA of short- (4 week) and long-term (12 week) sucrose consuming adolescent rats and compared these to age-matched water controls. Our results indicate possibly maladaptive changes to the dendritic architecture of BLA principal neurons, particularly on apical dendrites following long-term sucrose consumption. Specifically, our results show reduced total dendritic arbor length of BLA principal neurons following short- and long-term sucrose consumption. Additionally, we found that long-term binge-like sucrose consumption caused a significant reduction in the length and complexity of apical dendrites. Taken together, our results highlight the differences between short- and long-term binge-like sucrose consumption on BLA principal neuron morphology and are suggestive of a perturbation in the diverse synaptic inputs to these neurons.

## Introduction

Increased sugar intake is considered one of the fundamental and principal factors of the current worldwide obesity epidemic [1]. While a compelling body of evidence suggests that heightened consumption of sugar partly influences weight gain among adults [2] and more notably in children and adolescents [3, 4], recent studies suggest that high sugar consumption may also result in neural changes in brain regions involved in reinforcement and determining incentive salience of sweetened food [5, 6]. Indeed, consumption of sugar and sweetened food in humans can cause cravings similar to those produced by addictive substances such as alcohol, nicotine or cocaine, primarily by activating the mesolimbic reward pathway [7]. In addition, previous studies that have examined the effects of sucrose and diet-induced obesity on incentive and motivation mediated by the NAc and glutamatergic plasticity in the NAc, have shown that diets high in fat or sucrose enhance AMPA receptors in the NAc [8, 9]. Furthermore, other studies have shown enhanced striatal dopamine release in response to increased insulin [10], as well as the involvement of the NAc in response to highly palatable food types [11].

The mesolimbic reward pathway is a collection of highly interconnected brain nuclei including the nucleus accumbens (NAc), the ventral tegmental area (VTA) and the amygdala that encode emotional states such as anticipation of reward and motivation [12]. In relation to sugar consumption, this reward pathway has been shown to display an exaggerated incentive salience response to cues for sucrose [13–15]. There is also evidence that suggests long-term consumption of highly palatable food can cause adaptations in the brain reward pathways, suggestive of an imbalance in the normal reward processing homeostasis [6, 16, 17]. While we have previously shown that medium spiny neurons in the NAc undergo morphological changes following long-term sucrose consumption [18], other studies have also implicated the amygdala in incentive learning and motivational behaviors associated with the rewarding effects of addictive substances [19, 20]. In particular recent studies have highlighted the influence of the basolateral amygdala (BLA) to reward learning and the association with adaptive, goal-directed and emotional behavior [21].

In addition to afferents from the medial prefrontal cortex, thalamus and hippocampus [22–25], the BLA also receives dopaminergic input from the VTA [26]. Additionally, the BLA sends glutamatergic efferents to the medium spiny neurons in the NAc, a key region of the mesolimbic reward pathway [27–29]. It is suggested that synaptic connectivity between the BLA and NAc is critically involved in reward-seeking behavior [19]. These circuits may underlie behavioral changes due to drug addiction and therefore warrant a closer examination in relation to sucrose consumption. Given that our previous studies have shown significant changes in NAc neuron morphology following long-term binge-like sucrose consumption [18] as well as altered responsiveness in relation to the accumbal cholinergic tone due to prolonged sucrose consumption [30], we hypothesized that sucrose-consumption-mediated morphological changes may also occur in the BLA, a key region that facilitates reward-seeking behavior. We used Golgi-Cox staining to impregnate primary neurons from the BLA of short- (4 week) and long-term (12 week) 5% sucrose consuming rats on an intermittent-access paradigm and compared these to age-matched water controls. Our results indicate possibly maladaptive changes to the dendritic architecture of BLA principal neurons, particularly on apical dendrites following long-term sucrose consumption. Taken together, our results demonstrate the differences between short- and long-term binge-like sucrose consumption on BLA principal neuron morphology and are suggestive of an imbalance in the diverse inputs received by these neurons.

## Materials and methods

### Ethics statement

All experimental procedures were carried out in accordance with the Australian Code for the Care and Use of Animals for Scientific Purposes, 8th Edition (National Health and Medical Research Council, 2013). The protocols were approved by the Queensland University of Technology Animal Ethics Committee and the University of Queensland Animal Ethics Committee.

### Animals and housing

Five-week-old (adolescent) male wistar rats (Control: 176.5 ± 5.0 g; Sucrose: 178.1 ± 5.1 g) (ARC, WA, Australia), were individually housed in ventilated dual level Plexiglas^®^ cages. The rats were acclimatized to the individual housing conditions, handling, and reverse-light cycle 5 days before the start of the experiments. All rats were housed in a climate-controlled 12-hr reversed light/dark cycle (lights off at 9 a.m.) room with standard rat chow and water available *ad libitum* as described in detail previously [18, 30].

### Intermittent-access two-bottle choice drinking paradigm

The intermittent access 5% sucrose two-bottle choice drinking paradigm [30, 31] was adapted from [32]. All fluids were presented in 300 ml graduated plastic bottles with stainless-steel drinking spouts inserted through two grommets in the front of the cage following the commencement of the dark reverse-light cycle. Weights of each bottle were recorded prior to bottle presentation. Two bottles were presented simultaneously: one bottle containing water; the second bottle containing 5% (w/v) sucrose. To control for side preferences, the placement of the 5% (w/v) sucrose bottle was alternated on each exposure. Bottles were weighed 24 h after the fluids were presented, and measurements were taken to the nearest 0.1 g. The weight of each rat was also measured to calculate the grams of sucrose intake per kilogram of body weight. On day 1 of the drinking period, rats (*n* = 7) were given access to one bottle of 5% (w/v) sucrose and one bottle of water. After 24 h, the sucrose bottle was replaced with a second water bottle that was available for the next 24 h. This pattern was repeated on Wednesdays and Fridays. The rats had unlimited access to water on all other days and was accompanied by stable baseline drinking levels based on body weight [20 ± 5 g/kg of 5% (w/v) sucrose] during the short-term [~4 weeks (13 drinking sessions)] and long-term [~12 weeks (37 drinking sessions)] drinking periods. A separate group of control rats (*n* = 7) were given access to water in both bottles (i.e., no sucrose) under the same conditions described above. The mean body weight of control and sucrose consuming rats at the end of short-term exposure was 426.0 ± 36.9 g and 439.2 ± 24.7 g respectively. At the end of long-term exposure, the mean body weight for control and sucrose groups was 590.0 ± 58.2 g and 617.8 ± 36.4 g.

### Golgi-Cox staining

Golgi-Cox staining was performed as described previously [18]. Briefly, following the last drinking session, rats were sacrificed by sodium pentobarbital overdose (60–80 mg/kg, i.p. Vetcare, Brisbane, Australia) and intracardially perfused with ~300 ml artificial cerebro-spinal fluid that contained, (in mM): 130 NaCl, 3 KCl, 26 NaHCO_3_, 1.25 NaH_2_PO_4_, 5 MgCl_2_, 1 CaCl_2_, and 10 D-glucose. Brains were incubated in the dark in Golgi-Cox solution that contained 5% potassium dichromate, 5% potassium chromate, and 5% mercuric chloride (all chemicals from Sigma-Aldrich). Brains from short-term sucrose consuming animals were incubated for 6 days at 37°C, whilst brains from long-term sucrose consuming animals were incubated for 10 days, with one change to fresh Golgi-Cox solution after 4 days of incubation.

Following incubation, 300 μm coronal sections were cut using a vibrating Zeiss Hyrax V50 microtome (Carl Zeiss, Germany). Slices were then placed sequentially in 24-well plates filled with 30% (w/v) sucrose in 0.1 M phosphate buffered saline and processed as outlined previously [33]. The sections were then cleared in CXA solution (1:1:1 chloroform:xylene:alcohol) for 10 min and mounted in DPX (Sigma-Aldrich) on Superfrost Plus slides (Menzel-Glaser, Lomb Scientific, Australia) and cover-slipped (Menzel-Glaser, Germany). The slides were left in the dark to dry at room temperature overnight.

### Neuronal selection and tracing within the BLA

**Fig 1:**
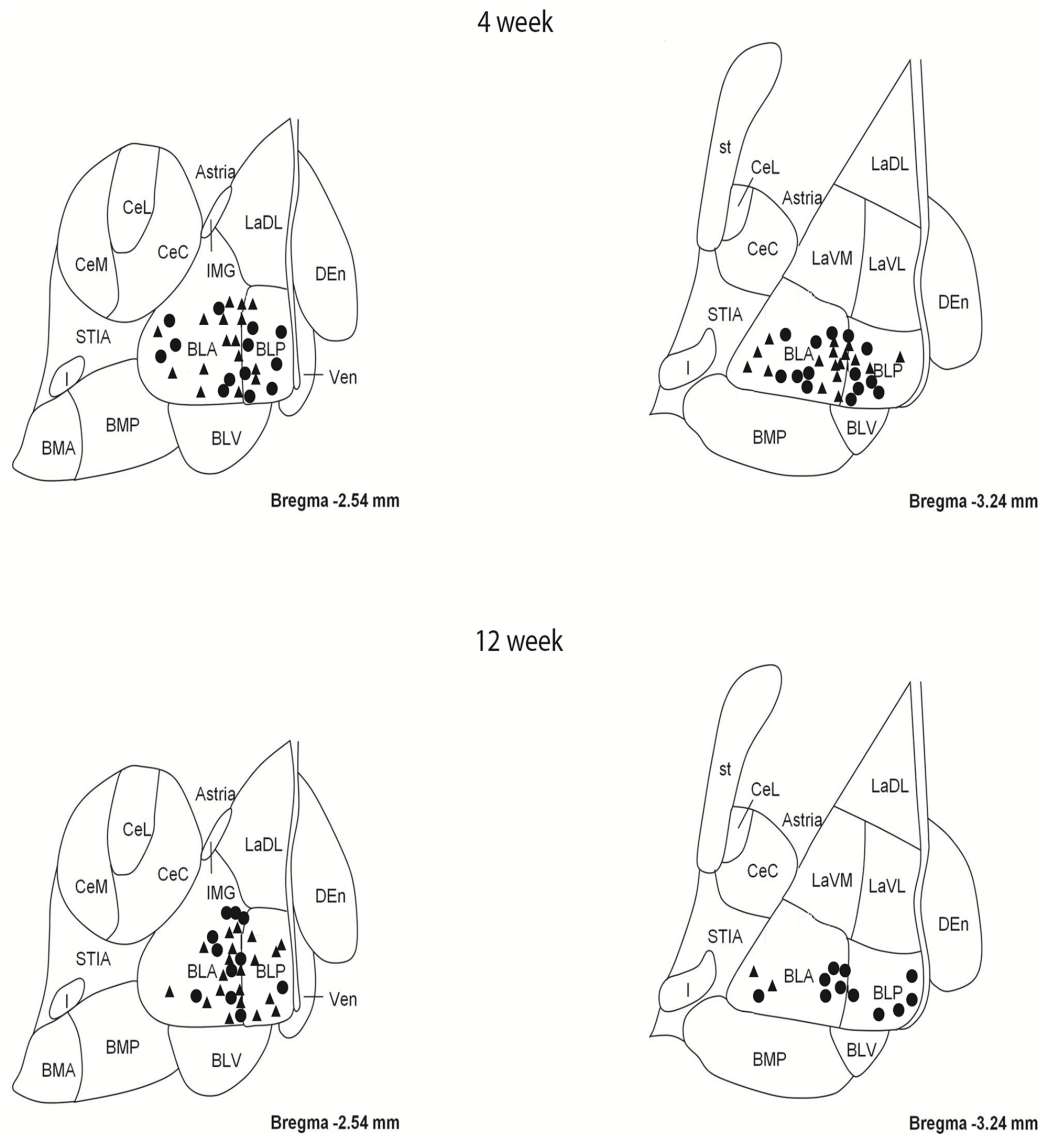
Map showing locations of principal neurons sampled from the basolateral amygdala (BLA) of 4 and 12 week sucrose consuming rats and age-matched controls. Top two panels show locations of neurons sampled from the BLA of 4 week control (triangles) and sucrose (circles) rats. Bottom two panels show locations of neurons sampled from the BLA of 12 week control (triangles) and sucrose (circles) rats.

As described in detail previously [18], coronal slices between bregma -2.54 and -3.24 were surveyed for principal neurons within the BLA, using the internal capsule and the external capsule as landmarks with the aid of a rat brain atlas [34]. The contour function in Neurolucida 7 (MBF Bioscience, VT, USA) was used to demarcate the BLA and the LA in each slice. Between 2 and 6 neurons were sampled from the anterior and posterior basolateral amygdaloid nuclei within the BLA from each animal (Fig 1) and were traced for dendritic length parameters using a 63x objective or for spine densities (reported as spines per 100 μm) using a 100x objective on a Zeiss Axioskop II (Carl Zeiss, Germany) using an automated *xyz* stage driven by Neurolucida^®^ 7 software (MBF Biosciences, VT, USA). All tracing was performed in a blinded fashion with respect to treatment. Morphological parameters of Golgi-Cox impregnated neurons were analyzed in a manner similar to previous reports [35].

### Statistical analysis

Mean and standard error of the mean (SEM) were calculated for each data set with the animal as *n*, using the mean morphometry data from all the BLA principle neurons (*n* = 7 for control and n = 7 for sucrose). Where indicated, unpaired two tailed Student's *t*-tests or two-way ANOVAs with Bonferroni post-tests were conducted for all analyses involving the comparison of group means, using GraphPad Prism version 6.02 (GraphPad Software, San Diego, CA). Statistical significance was accepted at *P*< 0.05. All data in the results section are presented as means ± SEM. Percentage changes are calculated as relative to the control value.

## Results

**Fig 2:**
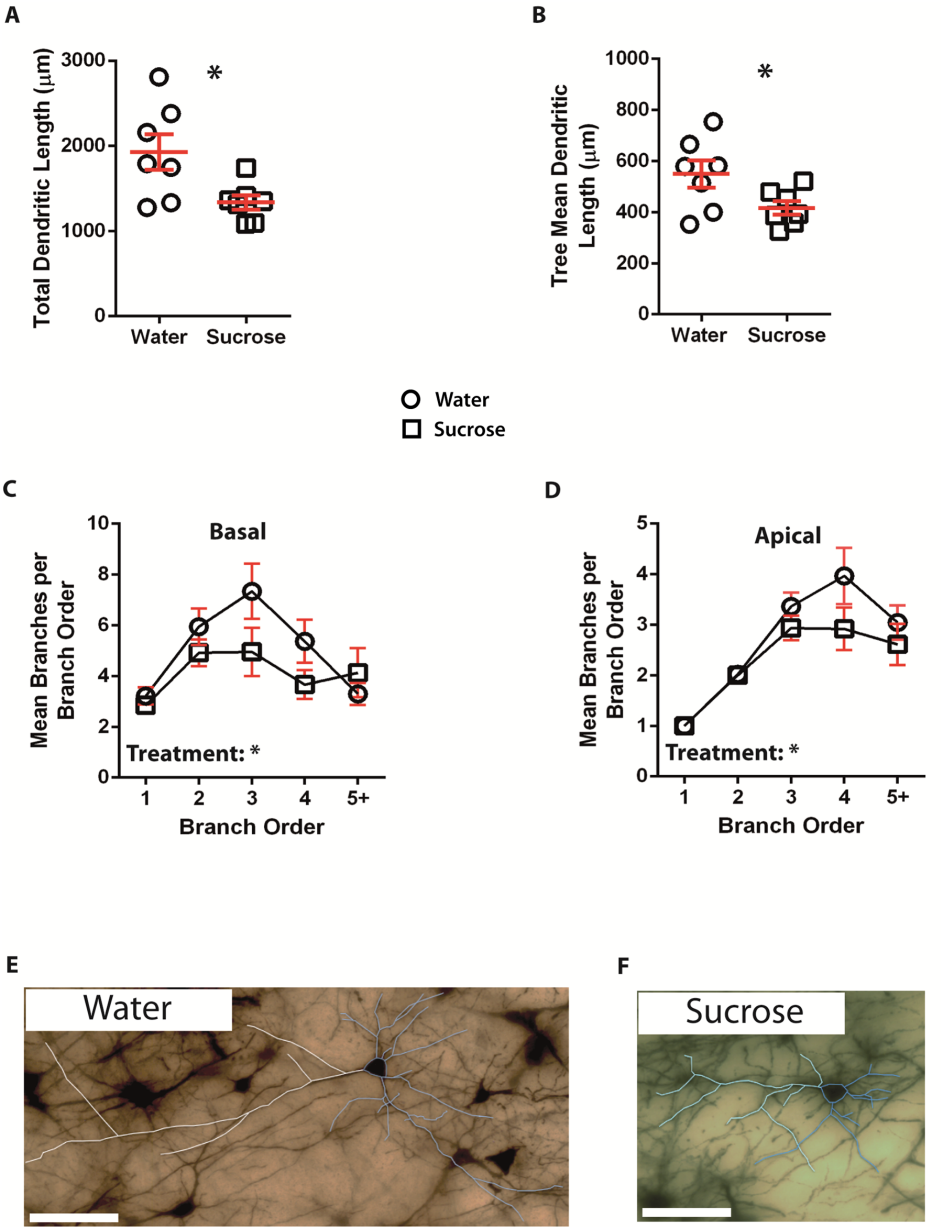
Decreased dendritic arbor length of principal neurons from the basolateral amygdala (BLA) of short-term sucrose consuming rats compared to control rats. **A** shows a scatter-plot of decreased total dendritic arbor (mean ± SEM) from the BLA in short-term sucrose rats (open squares) compared to controls (open circles) (Unpaired two-tailed Students *t*-test,*P < 0.05, *n* = 7; control and *n* = 7; 4 week sucrose). **B** shows a scatter-plot of decreased mean dendritic tree length (mean ± SEM) from the BLA in short-term sucrose rats (squares) compared to controls (circles) (Unpaired two-tailed Students *t*-test, *P < 0.05, *n* = 7; control and *n* = 7; 4 week sucrose). Branch order analysis (mean ± SEM) showing decreased number of dendritic segments per branch order for basal dendrites **(C)** and apical dendrites **(D)** from sucrose consuming rats (squares) and water control rats (circles) (two-way ANOVAs, *n* = 7; control and *n* = 7; short-term sucrose). **E and F** show representative brightfield *z*-stack mosaics of Golgi-Cox impregnated principal neurons from the BLA (63x magnification) of control (water) and short-term (4 week) sucrose drinking rats respectively. Scale Bars: **(E, F)** = 100 μm.

Following short-term (4 weeks) sucrose consumption, the total dendritic arbor length of principal neurons in the BLA was decreased by 31% compared to water consuming controls (Water: 1928 ± 211 μm, *n* = 7; Sucrose 1337 ± 84 μm, *n* = 7, *P* = *0.0229, two-tailed unpaired Student's *t*-test, Fig 2A, Table 1). Comparison of the mean number of dendritic bifurcations (nodes) and dendritic endings between the water and sucrose groups revealed a significantly reduced level of dendritic complexity in the principal neurons of the BLA (nodes: Water 9.4 ± 1.2 *n* = 7, Sucrose 5.7 ± 1.0 *n* = 7, *P* = *0.0349; endings: Water 11.4 ± 1.2 *n* = 7, Sucrose 7.7 ± 1.0 *n* = 7, *P* = *0.0385, two-tailed unpaired Student's *t*-test, Table 1). Also, mean dendritic tree length was significantly reduced in the sucrose group compared to the water consuming controls (Water 550 ± 53 *n* = 7, Sucrose 417 ± 26 *n* = 7, *P* = *0.0451, Fig 2B, Table 1). There was no change in total spine density (*P* = 0.1353). These morphometric parameters are detailed in Table 1, and graphically represented in Fig 2 (Sucrose—open circles; Control—open squares).

**Table 1:** General morphologic parameters of principal neurons from the BLA of short-term sucrose consuming rats and age-matched water controls.

Parameter	Water (n)	Sucrose (n)	*P*-value
Total dendritic length (μm)	1928 ± 211 (7)	1337 ± 83 (7)	0.0229*
Mean tree length (μm)	550 ± 53 (7)	417 ± 26 (7)	0.0451*
Basal	391 ± 35 (7)	286 ± 23 (7)	0.028*
Apical	709 ± 98 (7)	547 ± 45 (7)	0.1614
Nodes	9.4 ± 1.2 (7)	5.7 ± 1 (7)	0.0349*
Basal	12 ± 1.5 (7)	7.3 ± 1.6 (7)	0.0511
Apical	6.8 ± 1.2 (7)	4.2 ± 0.7 (7)	0.0827
Endings	11.4 ± 1.2 (7)	7.7 ± 1 (7)	0.0385*
Basal	15.1 ± 1.6 (7)	10.3 ± 1.7 (7)	0.0569
Apical	7.7 ± 1.2 (7)	5.2 ± 0.7 (7)	0.0887
Spines Per 100 μm	44.3 ± 2.9 (7)	49.9 ± 2 (7)	0.1353
Basal	44.4 ± 3.6 (7)	50.8 ± 1.7 (7)	0.1308
Apical	44.3 ± 3.2 (7)	49 ± 3.1 (7)	0.3046

(*: p<0.05, two-tailed unpaired Student's t-test)

**Fig 3:**
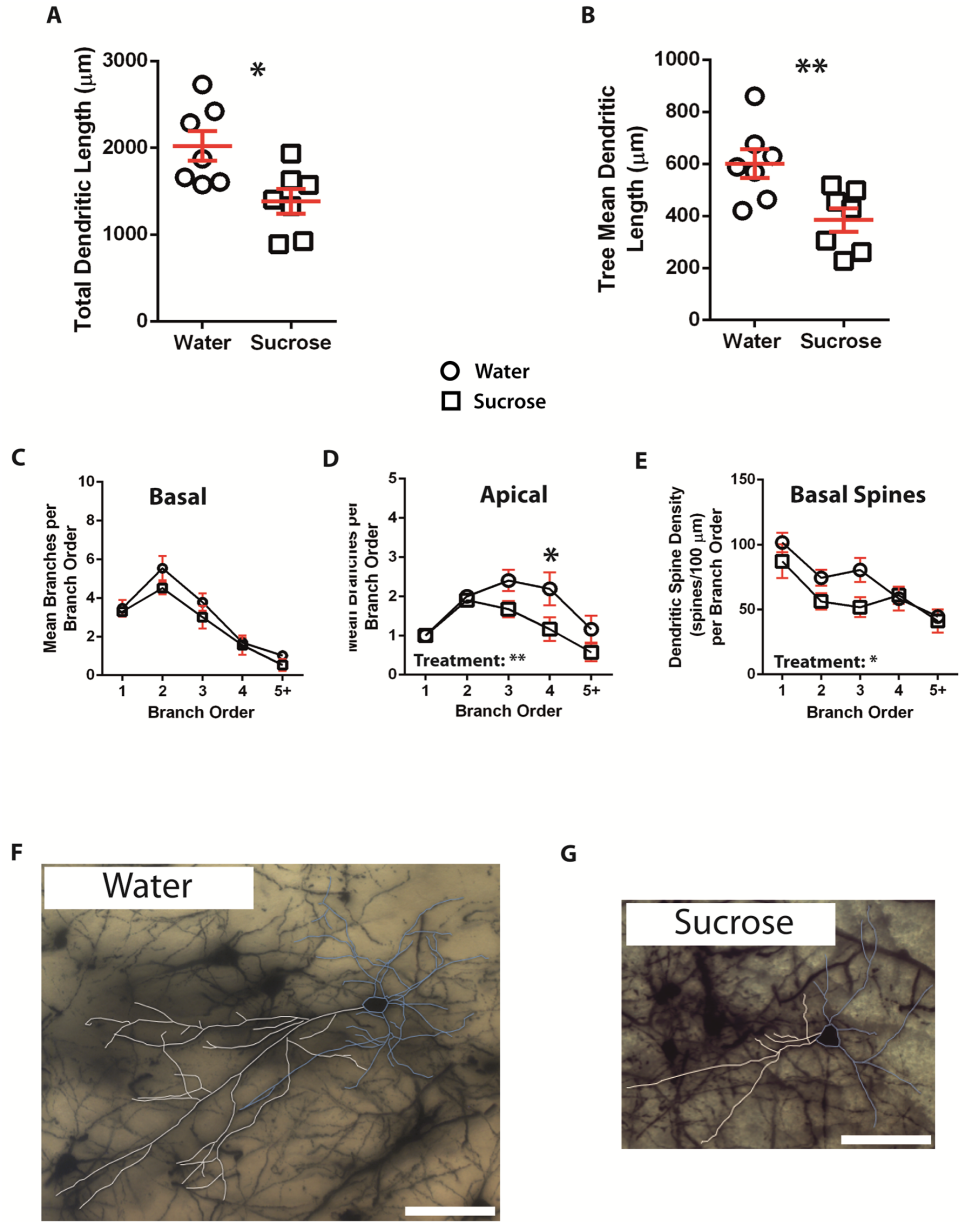
Decreased dendritic arbor length and distal branch number of principal neurons from the basolateral amygdala (BLA) of long-term sucrose consuming rats compared to control rats. **A** shows a scatter-plot of decreased total dendritic arbor (mean ± SEM) from the BLA in long-term sucrose rats (open squares) compared to controls (open circles) (Unpaired two-tailed Students *t*-test,*P < 0.05, *n* = 7; control and *n* = 7; 12 week sucrose). **B** shows a scatter-plot of decreased mean dendritic tree length (mean ± SEM) from the BLA in long-term sucrose rats (squares) compared to controls (circles) (Unpaired two-tailed Students *t*-test, **P < 0.01, *n* = 7; control and *n* = 7; 12 week sucrose). Branch order analysis (mean ± SEM) showing no change in the number of dendritic segments for basal dendrites **(C),** but decreased dendritic segment number for apical dendrites from sucrose drinking rats (square) compared to water controls (triangles) **(D**, two-way ANOVA). **E** shows decreased dendritic spine density per branch order for basal dendrites from long-term sucrose rats compared to controls (two-way ANOVA). Bonferroni post-tests revealed a significant reduction in the apical segment number at distal 4^th^ order branches in long-term sucrose consuming rats compared to controls **(D)** (two-way ANOVA with Bonferroni post-tests,*P < 0.05, *n* = 7; control and *n* = 7; long-term sucrose). **F and G** show representative brightfield *z*-stack mosaics of Golgi-Cox impregnated principal neurons from the BLA (63x magnification) of control (water) and long-term (12 week) sucrose drinking rats respectively. Scale Bars: **(F, G)** = 100 μm.

Following long-term (12 weeks) sucrose consumption, the total dendritic arbor length of principal neurons in the BLA was decreased by 32% compared to water consuming controls (Water: 2023 ± 173 μm, *n* = 7; Sucrose 1384 ± 143 μm, *n* = 7, *P* = 0.0146, two-tailed unpaired Student's *t*-test, Fig 3A, Table 2). Comparison of the mean number nodes and dendritic endings showed reduced dendritic complexity in BLA principal neurons from the sucrose group compared to water controls (nodes: Water 5.0 ± 0.2 *n* = 7, Sucrose 3.8 ± 0.3 *n* = 7, *P* = **0.0032; endings: Water 7.4 ± 0.2 *n* = 7, Sucrose 5.9 ± 0.4 *n* = 7, *P* = **0.0042, two-tailed unpaired Student's *t*-test, Table 2). Further analysis revealed that dendritic complexity was significantly reduced in the apical but not basal dendrites of BLA principal cells (Apical nodes: Water 4.1 ± 0.5 *n* = 7, Sucrose 2.7 ± 0.4 *n* = 7, *P* = *0.0338; Apical endings: Water 5.1 ± 0.5 *n* = 7, Sucrose 3.7 ± 0.4 *n* = 7, *P* = *0.0416; Basal nodes: Water 5.9 ± 0.4 *n* = 7, Sucrose 4.9 ± 0.7 *n* = 7, *P* = 0.2095; Basal endings: Water 9.6 ± 0.8 *n* = 7, Sucrose 8.2 ± 0.8 *n* = 7, *P* = 0.2109, two-tailed unpaired Student's *t*-test, Table 2). Also, the mean dendritic tree length was significantly reduced in the sucrose group compared to the water consuming control (Water 601 ± 55 *n* = 7, Sucrose 385 ± 45 *n* = 7, *P* = **0.0099, Fig 3B, Table 2). Further analysis revealed a significant reduction in the mean dendritic tree length of BLA apical dendrites from sucrose consuming rats compared to controls (Water 847 ± 123 *n* = 7, Sucrose 504 ± 87 *n* = 7, *P* = *0.0423, Table 2). A trend towards reduced mean dendritic tree length of basal dendrites was also observed in the sucrose group compared to the control group (Water 355 ± 36 *n* = 7, Sucrose 266 ± 20 *n* = 7, *P* = 0.0516, Table 2). Total spine densities (*P* = 0.3171) of BLA principal neurons from long-term sucrose consuming rats were not different compared to the water controls. These morphometric parameters are detailed in Table 2, and graphically represented in Fig 3 (Sucrose—open circles; Control—open squares). Representative images of BLA principal neuron architecture are depicted in Figs 2E and 2F & 3F and 3G.

**Table 2:** General morphologic parameters of principal neurons from the BLA of long-term sucrose consuming rats and age-matched water controls.

Parameter	Water (n)	Sucrose (n)	*P*-value
Total dendritic length (μm)	2023 ± 173 (7)	1384 ± 143 (7)	0.0146*
Mean tree length (μm)	601 ± 55 (7)	385 ± 45 (7)	0.0099**
Basal	355 ± 36 (7)	266 ± 20 (7)	0.0516
Apical	847 ± 123 (7)	504 ± 87 (7)	0.0423*
Nodes	5 ± 0.2 (7)	3.8 ± 0.3 (7)	0.0032**
Basal	5.9 ± 0.4 (7)	4.9 ± 0.7 (7)	0.2095
Apical	4.1 ± 0.5 (7)	2.7 ± 0.4 (7)	0.0338*
Endings	7.4 ± 0.2 (7)	5.9 ± 0.4 (7)	0.0042**
Basal	9.6 ± 0.8 (7)	8.2 ± 0.8 (7)	0.2109
Apical	5.1 ± 0.5 (7)	3.7 ± 0.4 (7)	0.0416*
Spines Per 100 μm	77.3 ± 6.5 (7)	66.8 ± 7.8 (7)	0.3171
Basal	80.2 ± 7.4 (7)	65 ± 7.6 (7)	0.1753
Apical	74.5 ± 5.8 (7)	68.6 ± 8.4 (7)	0.5781

(*: p<0.05, **: p<0.01, two-tailed unpaired Student's t-test)

Subsequent to the analysis of the short-term and long-term dendritic morphology of sucrose consuming principal neurons in the BLA, we examined the dendritic arborizations and spine densities in relation to branch order. Our analysis assessed the number of dendritic segments per branch order, the mean length of dendritic segments per branch order and mean spine density per branch order of the basal and apical dendrites of the BLA principal neurons from short- and long-term sucrose consuming rats compared to their age-matched controls. Results are summarised in Tables 3–6 and described below.

**Table 3:** Branch order characteristics of basal dendrites from short-term sucrose and water drinking rats.

Branch Order Properties	Water (7)	Sucrose (7)	Adjusted P-value
1st order branch segments	3.2 ± 0.3	2.8 ± 0.1	> 0.9999
1st order mean branch segment length (μm)	33.5 ± 3.4	49.6 ± 7.4	0.5256
1st order branch spine density	54.5 ± 7.1	59.3 ± 3.3	> 0.9999
2nd order branch segments	5.9 ± 0.7	4.9 ± 0.5	> 0.9999
2nd order mean branch segment length (μm)	61.6 ± 9.6	54.3 ± 8.8	> 0.9999
2nd order branch spine density	58.2 ± 8.0	45.1 ± 6.8	0.6393
3rd order branch segments	7.3 ± 1.1	5.0 ± 1.0	0.1119
3rd order mean branch segment length (μm)	47.7 ± 4.8	48.4 ± 6.7	> 0.9999
3rd order branch spine density	42.8 ± 4.8	36.8 ± 6.4	> 0.9999
4th order branch segments	5.4 ± 0.8	3.7 ± 0.6	0.5051
4th order mean branch segment length (μm)	37.3 ± 5.7	45.2 ± 9.7	> 0.9999
4th order branch spine density	23.3 ± 7.1	35.7 ± 4.1	0.7493
5th order branch segments	3.3 ± 0.4	4.1 ± 1.0	> 0.9999
5th order mean branch segment length (μm)	35.4 ± 5.2	40.2 ± 4.4	> 0.9999
5th order branch spine density	21.0 ± 6.9	22.7 ± 3.5	> 0.9999

**Table 4:** Branch order characteristics of apical dendrites from short-term sucrose and water drinking rats.

Branch Order Properties	Water (7)	Sucrose (7)	Adjusted P-value
1st order branch segments	1.0 ± 0.0	1.0 ± 0.0	> 0.9999
1st order mean branch segment length (μm)	24.3 ± 2.8	57.3 ± 15.8	0.0926
1st order branch spine density	58.1 ± 8.9	54.2 ± 5.1	> 0.9999
2nd order branch segments	2.0 ± 0.0	2.0 ± 0.0	> 0.9999
2nd order mean branch segment length (μm)	69.5 ± 11.3	92.6 ± 17.5	0.4783
2nd order branch spine density	47.0 ± 4.5	43.4 ± 2.1	> 0.9999
3rd order branch segments	3.4 ± 0.3	2.9 ± 0.2	> 0.9999
3rd order mean branch segment length (μm)	65.4 ± 4.4	58.2 ± 4.7	> 0.9999
3rd order branch spine density	33.8 ± 4.6	38.9 ± 7.5	> 0.9999
4th order branch segments	4.0 ± 0.6	2.9 ± 0.4	0.0829
4th order mean branch segment length (μm)	38.1 ± 6.0	43.7 ± 9.2	> 0.9999
4th order branch spine density	38.3 ± 6.4	48.0 ± 7.7	> 0.9999
5th order branch segments	3.0 ± 0.3	2.6 ± 0.4	> 0.9999
5th order mean branch segment length (μm)	46.6 ± 7.2	38.3 ± 4.9	> 0.9999
5th order branch spine density	36.1 ± 2.0	50.2 ± 1.3	0.3913

**Table 5:** Branch order characteristics of basal dendrites from long-term sucrose and water drinking rats.

Branch Order Properties	Water (7)	Sucrose (7)	Adjusted P-value
1st order branch segments	3.5 ± 0.4	3.3 ± 0.2	> 0.9999
1st order mean branch segment length (μm)	49.1 ± 4.8	60.8 ± 7.9	> 0.9999
1st order branch spine density	101.7 ± 7.6	87.2 ± 13.0	> 0.9999
2nd order branch segments	5.5 ± 0.6	4.5 ± 0.3	0.4317
2nd order mean branch segment length (μm)	88.0 ± 5.6	67.8 ± 11.1	0.5964
2nd order branch spine density	74.5 ± 6.1	56.2 ± 6.2	0.583
3rd order branch segments	3.8 ± 0.5	3.0 ± 0.6	0.951
3rd order mean branch segment length (μm)	69.6 ± 7.2	64.7 ± 11.2	> 0.9999
3rd order branch spine density	80.5 ± 9.4	51.7 ± 7.6	0.074
4th order branch segments	1.7 ± 0.2	1.6 ± 0.5	> 0.9999
4th order mean branch segment length (μm)	39.6 ± 10.2	21.0 ± 7.7	0.7456
4th order branch spine density	58.4 ± 9.2	61.1 ± 6.6	> 0.9999
5th order branch segments	1.0 ± 0.2	0.5 ± 0.3	> 0.9999
5th order mean branch segment length (μm)	27.8 ± 9.6	19.7 ± 11.8	> 0.9999
5th order branch spine density	44.6 ± 1.0	41.2 ± 8.9	> 0.9999

**Table 6:** Branch order characteristics of apical dendrites from long-term sucrose and water drinking rats.

Branch Order Properties	Water (7)	Sucrose (7)	Adjusted P-value
1st order branch segments	1.0 ± 0	1.0 ± 0	> 0.9999
1st order mean branch segment length (μm)	43.6 ± 8.4	45.0 ± 16.2	> 0.9999
1st order branch spine density	117.3 ± 8.3	123.0 ± 15.0	> 0.9999
2nd order branch segments	2.0 ± 0	1.9 ± 0.2	> 0.9999
2nd order mean branch segment length (μm)	104.1 ± 22.3	78.8 ± 17.4	> 0.9999
2nd order branch spine density	70.0 ± 6.7	70.9 ± 9.2	> 0.9999
3rd order branch segments	2.4 ± 0.3	1.7 ± 0.2	0.1856
3rd order mean branch segment length (μm)	78.7 ± 12.9	67.2 ± 18.5	> 0.9999
3rd order branch spine density	77.2 ± 7.7	58.2 ± 7.3	0.6421
4th order branch segments	2.2 ± 0.4	1.2 ± 0.3	0.0191*
4th order mean branch segment length (μm)	51.2 ± 14.7	33.1 ± 10.5	> 0.9999
4th order branch spine density	78.3 ± 5.6	61.2 ± 6.6	0.8579
5th order branch segments	1.2 ± 0.3	0.6 ± 0.2	0.4459
5th order mean branch segment length (μm)	54.6 ± 16.0	16.7 ± 7.4	0.4058
5th order branch spine density	80.5 ± 11.1	64.6 ± 5.4	> 0.9999

(*: p<0.05, two-way ANOVA with Bonferroni *post-hoc* analysis)

The mean dendritic branch segment number per branch order of BLA principal neuron apical dendrites significantly decreased in short-term and long-term sucrose consuming rats compared to water controls (4-weeks: *P* = *0.0467, two-way ANOVA. 12 weeks: *P* = **0.0022, two-way ANOVA, Figs 2 and 3). Bonferroni post-tests revealed a non-significant decrease in the number of branch segments at 4^th^ order branches (Water: 4.0 ± 0.6, *n* = 7; Sucrose 2.9 ± 0.4, *n* = 7, *P* = 0.0829, Fig 2D, Table 4) in short-term sucrose rats, while a significant reduction in branch segments at 4^th^ order branches (Water: 2.2 ± 0.4, *n* = 7; Sucrose 1.2 ± 0.3, *n* = 7, *P* = *0.0191, Fig 3D, Table 6) was found in BLA principal cell apical dendrites from long-term sucrose rats compared to water controls. The mean dendritic branch segment number per branch order of BLA principal neuron basal dendrites was significantly decreased in short-term sucrose consuming rats (*P* = *0.046, two-way ANOVA, Fig 2C) and a trend to a reduction was observed in long-term sucrose consuming rats compared to water controls (*P* = 0.053, two-way ANOVA, Fig 3C).

The mean dendritic segment length per branch order of BLA principal neurons showed a trend towards reduced branch segment length in long-term sucrose consuming rats compared to water controls (*P* = 0.0604, two-way ANOVA) in apical dendrites. Furthermore, branch order analysis showed a significant decrease in dendritic spine density in the basal dendrites of BLA principal neurons of long-term sucrose consuming rats compared to controls (*P* = *0.0183 two-way ANOVA, Fig 3E). Bonferroni post-tests revealed a non-significant trend towards reduced spine density at distal 3^rd^ order branches (Water: 80.5 ± 9.4, *n* = 7; Sucrose 51.7 ± 7.6, *n* = 7, *P* = 0.074, Table 5).

Lastly, comparison of short-term (4 weeks) and long-term (12 weeks) control groups reveal no significant differences in the total dendritic arbor length or mean tree length, either in the basal or apical dendrites. There was, however, a significant decrease in nodes (both basal and apical) and endings (only basal). Furthermore, there was a significant increase in spine density in the 12-week control group as compared to the 4-week control group, in both the basal and apical dendrites. Analysis of branch order characteristics revealed a significant increase in branch segment length and spine density concomitant with a significant decrease in number of branch segments (3^rd^ branch order and above) of long-term (12 week) controls compared to short-term (4 week) controls, in both the basal and apical dendrites.

Taken together, results from our present study indicate that short-term binge-like sucrose consumption has a significant effect on the general morphology parameters of principal neurons in the BLA. Additional changes, particularly in apical dendrites, are also observed in BLA principal neurons from long-term sucrose consuming rats compared to age-matched controls. Furthermore, branch structure analysis revealed a reduced number of apical and distal 4^th^ order branches in long-term sucrose consuming rats. In contrast, the morphological parameters of basal dendrites were not as responsive to the effects of short- and long-term sucrose consumption, although, we did observe an overall reduction in spine density in basal dendrites when analysed with respect to branch order.

## Discussion

Obesity and its related pathologies are showing increasing trends worldwide. While the contribution of genetics, epigenetics as well as socio-economic factors towards weight gain cannot be understated, several studies have shown a positive association between weight gain and the consumption of sugar-sweetened food and beverages [3, 36–40], particularly in children [41–43]. Interestingly, in humans, the consumption of sugar and sweetened food can cause cravings similar to those produced by addictive substances such as alcohol, nicotine or cocaine, primarily via activation of the mesolimbic reward pathway [7]. We therefore used a model of binge-like sucrose consumption in rats to determine the effects of short- (4 weeks) and long-term (12 weeks) intake on neuronal morphology of principal neurons in the BLA, a key component of the overlapping reward circuitry that is also modulated by addictive drugs. We show that principal neurons from the BLA of short- and long-term sucrose consuming rats have significantly decreased dendritic length and complexity. Surprisingly, we found that the total spine densities of BLA principal cells were not affected by binge-like consumption of sucrose. Overall, these findings demonstrate the contribution of long-term binge-like sucrose intake on neuronal morphology of the BLA principal neurons and also underscore the possibly detrimental effects of diets containing excess amounts of sugar.

The BLA, which forms part of the amygdala, is comprised mainly of principal neurons, which are morphologically characterized by a pyramidal (or piriform) cell body with a “thick” apical dendrite and “thinner” basal dendrites [44–48]. These pyramidal cells form ~80% of the population of neurons in the BLA, the remainder of which are comprised of GABAergic interneurons. The generation of emotional responses in the BLA is largely dependent on the balance of excitatory and inhibitory afferents to the primary neurons [49], with the GABAergic interneurons providing tight regulation of the inhibitory tone within the BLA [50, 51]. The BLA plays an important role in adaptive, goal-directed behavior [21], and is critical for the production of directed emotional responses and the processing of emotional memories [52]. Furthermore, synaptic connectivity between the BLA and NAc has been suggested to be essential in reward-seeking behavior [19]. Given that BLA principal cell apical dendrites receive distal glutamatergic inputs that play a pivotal role in the formation of emotional and associative memories, it is interesting to note that our study revealed significant restructuring of apical distal branches. A change in the level of excitatory inputs received by BLA principal neurons is likely to influence their neuronal activity. Whether long-term sucrose induced reductions in dendritic length and branching of BLA principal neurons, particularly in the distal apical dendrites are associated with reductions in their excitatory activity and neuronal output is an interesting possibility that warrants further investigation.

In relation to morphological changes of BLA neurons, various studies have examined the effect of drug-intake. For example, [53] demonstrated that chronic nicotine exposure produces age-dependent dendritic remodeling in the rodent basolateral amygdala (also reviewed here—[54]). Also, evidence suggests that prenatal ethanol exposure alters dendritic morphology in the BLA of rat offspring [55]. Bergstrom and colleagues (2010) showed that chronic nicotine exposure reduced dendritic complexity, a finding similar to our results. In contrast, they reported increased total dendritic length, an observation also noted in chronic alcohol exposure [55]. In addition, chronic immobilization stress (CIS) has also shown to induce greater total dendritic length concomitant with enhanced dendritic arborisation [56]. Therefore, it is possible that the observed alteration to dendritic morphology seen in our study could mirror the effects seen with drugs of abuse. Indeed, our previous study that examined the effects of sucrose consumption in the NAc [18] identified lower total dendritic length and increased distal spine density, hallmarks of morphological changes seen in response to drugs of abuse such as alcohol, cocaine and amphetamines. Interestingly, studies have also shown that not only drugs of abuse but also a diet high in fat attenuates dendritic spine density in regions mediating reward and motivation [57] as well as decreased inhibitory synaptic transmission [58] in the lateral orbitofrontal cortex that is part of the prefrontal cortex, a key region intricately involved with processing reward and motivation. Taken together, this suggests the possibility of overlapping neural pathways for drugs of abuse and certain foods types. Additionally, lower dendritic complexity as well as reduced total dendritic length, both findings observed in our study, are noted in reversal of stress-induced anxiety [59], suggesting that dendritic hypotrophy in the BLA may act as a resilience marker [60]. While the changes observed in our study resemble some changes due to drugs of abuse, the absence of changes in spine density in our study, particularly at distal dendritic branches suggest that additional changes are specific to high sucrose intake. In particular, altered neuronal pruning could be an additional factor that may play a role in the observed changes to the morphometry of BLA principal neurons, especially following long-term binge-like sucrose consumption. It is also noteworthy that exposure to various drugs of abuse as well as various types of stress can cause an increase in distal spine density in the BLA principal neurons [61, 62]. In contrast, studies with fatty acid amide hydrolase (FAAH) deficient rodents are marked by an absence in spine density change by those very same stressors [63], but coupled with an increased sucrose consumption [64]. This inverse relationship between increased sucrose consumption and reduced function of FAAH, and the concomitant absence of spines in FAAH deficient paradigms, may provide an explanation for the absence of spine density changes seen in our study. However, further studies are required to investigate these effects in more detail.

Lastly, It is noteworthy to take into consideration that the present study’s experimental model utilised rodents that started consuming sugar in adolescence. Other studies that have examined neuronal morphological parameters, found changes in response to food as well, albeit in adults [57]. Furthermore, enhanced excitability of NAc medium spiny neurons in response to diet-induced obesity has been noted in adult but not adolescent rats [8]. It is particularly relevant to note that sucrose consumption increases synaptic abundance of AMPA receptors in nucleus accumbens in adults [9]. Taken together, these studies, in conjunction with our present study, highlight the importance of studying critical periods of development in terms of neuron development and function.

In conclusion, although further functional studies are needed to ascertain the mechanisms underpinning the morphological changes seen in our study, these results demonstrate significant neuronal effects due to binge-like sucrose consumption at the level of the BLA. Given the importance of the BLA in encoding emotional salience and its involvement in adaptive goal-directed behaviours especially via afferents to the NAc, our study highlights the importance of examining the effects of excessive sugar consumption (particularly following long-term intake) on brain regions that modulate the reward circuitry, in order to provide a greater understanding of the neuronal effects contributing to the obesity epidemic and its attendant morbidities.
